# Diversity of SpaP in genetic and salivary agglutinin mediated adherence among *Streptococcus mutans* strains

**DOI:** 10.1038/s41598-019-56486-9

**Published:** 2019-12-27

**Authors:** Jingmei Yang, Dongmei Deng, Bernd W. Brandt, Kamran Nazmi, Yafei Wu, Wim Crielaard, Antoon J. M. Ligtenberg

**Affiliations:** 1 State Key Laboratory of Oral Diseases & National Clinical Research Center for Oral diseases & Department of Periodontics, West China School & Hospital of Stomatology, Sichuan University, Chengdu, China; 2 Department of Preventive Dentistry, Academic Centre for Dentistry Amsterdam, University of Amsterdam and Vrije Universiteit Amsterdam, Amsterdam, The Netherlands; 3 Department of Oral Biochemistry, Academic Centre for Dentistry Amsterdam, University of Amsterdam and Vrije Universiteit Amsterdam, Amsterdam, The Netherlands

**Keywords:** Cell adhesion, Bacteriology

## Abstract

*Streptococcus mutans* SpaP mediates the binding of this cariogenic bacteria to tooth surfaces. It was reported that the SpaP of *S. mutans* clinical isolates could be classified to 2 genotypes, type A and B. Our aims are to examine *spaP* genotypes in often-used *S. mutans* laboratory strains as well as clinical isolates and to explore the relationship between the genotypes of *S. mutans* strains and their adherence to salivary-agglutinin (SAG). The sequences of SpaP of 11 *S. mutans* strains were analyzed with alignment tools. Out of these strains, 9 strains were examined for their adherence to SAG-coated surfaces. The SpaP expression on the cell surfaces and in the spent media of 9 strains were examined by a dot-blot assay. Based on the alignment of the variable V region of SpaP, 9 strains were classified as previously-defined type-A and 3 strains type-B. Among type-B strains, the SpaPs of GS5 and HG723 contain a premature stop codon which resulted in loss of adherence and absence of SpaP expression on the cell surfaces. However, clear SpaP expression was observed in the spent media of both strains. The type-B strain UA159 demonstrated low SpaP expression on the cell surface, but it showed similar adherence ability as the type-A strains. In conclusion, the presence of SpaP on the cell surface determines the adherence of *S. mutans* to SAG. No difference in SAG-mediated adherence could be seen between type A and B strains, probably due to the limited number of type B strain tested.

## Introduction

*Streptococcus mutans* has been recognized as the principal bacterial agent of dental caries^1^. Next to its acidogenic potential, its ability to adhere to teeth and form a biofilm contributes to its cariogenicity^2^. One crucial adhesion and colonization factor of *S. mutans* is a conserved sucrose-independent adhesin, SpaP^3,4^. SpaP is also named P1, Antigen I/II or Pac^3^. It mediates the adherence of *S. mutans* to the saliva-coated tooth surface by interacting specifically with a salivary component, salivary agglutinin (SAG)^5^. SAG, also known as glycoprotein-340 (gp 340) or SALSA, is encoded by the gene Deleted in Malignant Brain Tumours 1 (DMBT1)^6^. The protein is characterized by multiple scavenger receptor cysteine rich (SRCR) domains separated by scavenger interspersed domains with potential O-glycosylation sites. SpaP recognizes conserved peptide sequences of the SRCR domain and possibly carbohydrates^3,7,8^.

Previous studies on the structure of the *S. mutans* SpaP protein revealed that this protein comprises a leader peptide (aa residues 1–38) adjacent to a series of alanine-rich (A) repeats (aa residues 186–464), a variable (V) region (aa residues 465–839), a series of proline-rich (P) repeats (aa residues 840–963) and an LPXTG cell-wall anchor motif ^9^. Larson *et al*.^10^ reported the crystal structure of the A_3_VP_1_ (a stalk-like structure formed by the third A region repeat and the first P region repeat while presenting a V region head) fragment of *S. mutans* SpaP representing a functional structure for adherence. Due to the critical role of SpaP in the SAG-mediated *S. mutans* adherence, this protein has become a promising candidate for developing protective immunization strategies against *Streptococcus* infection^3^.

SpaP is widely distributed throughout the streptococci. Although the orthologous proteins generally have a conserved primary structure with 70–90% sequence similarity^3^, the function of the protein can vary in different streptococci^4,11^. This variation is possibly caused by the multi-functional nature of the SpaP protein family. The SpaP protein consists not only of the binding site for SAG, but also sites with affinities for fibronectin, collagen and other bacterial species like *P. gingivalis*^3^. Hence, the combination of functions of SpaP can be different in different streptococci^11^.

However, the sequences of *S. mutans* SpaPs are highly conserved with around 90% sequence similarity^12^. It is believed that the variable V-region determines the diversity of SpaP among *S. mutans* strains. Recent studies reported that *S. mutans* SpaPs can be grouped into two types (A and B) based on the variable V-region segments^12,13^. *S. mutans* strains with SpaP type B showed a much stronger binding to SAG than those with type-A^13^ and the presence of the type-B strains could be associated with an increase in caries over a 5-year period^12^.

Until now, laboratory strains of *S. mutans* have been used to examine the function of SpaP^14^, while their *spaP* genotypes were unknown. In order to better understand the involvement of SpaP in the virulence of *S. mutans*, it is important to know the *spaP* genotypes of both well-known laboratory strains and clinical isolates. Hence, the aims of our study are to examine the *spaP* genotypes in the known *S. mutans* laboratory strains as well as our own clinical isolates and to explore the relationship between the genotypes and *S. mutans* adherence to SAG-coated surfaces.

## Results

### Comparison of SpaP protein sequences among *S. mutans* strains

**Figure 1:**
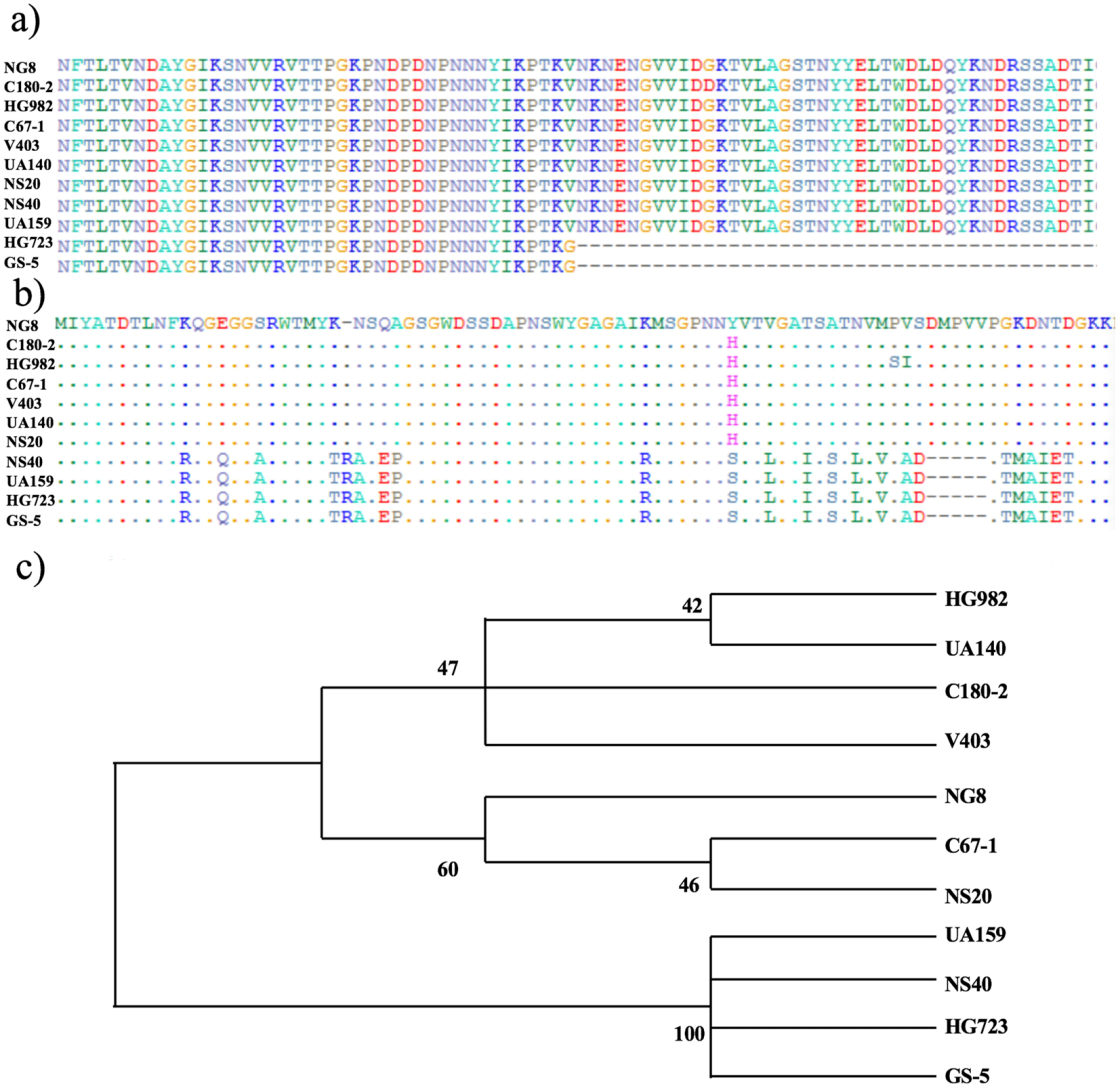
SpaP protein sequence alignment and phylogenetic tree. In (**a**), the region (residues 1123–1204) that contains the premature stop codon of the full protein sequence alignment of SpaP is shown. HG723 and GS-5 have a premature stop codon after 1158 residues. (**b**) Shows the alignment of the most variable section A_3_VP_1_ (residues 371–823) among 11 *S. mutans* strains. (**c**) Shows the phylogenetic tree based on this region, indicating that 11*S. mutans* strains were grouped into two clusters.

The alignment of the 11 complete protein sequences are shown in Fig. 1a). The SpaP sequences of strains HG723 and GS-5 are identical. Both terminate after 1158 amino acids: in their corresponding nucleotide acid sequences, a single base (adenine) was inserted at position 3470, which resulted in the frame shift and premature stop codon. Figure 1a) shows the corresponding region of the complete alignment. Consequently, the length of the proteins in these two strains is 1158 amino acids, much shorter than those of the other 9 *S. mutans* strains. Among 9 strains, the length of SpaP of strains UA159 and Nicklas Stromberg 40 is 1562, 4 amino acids shorter than that of the other 7 strains (1566). The *spaP* nucleotide acid sequence of strain UA159 fully matches the published *spaP* sequence (Gene ID: 1028055).

Since the region of A_3_VP_1_ was shown to mediate the high-affinity binding of SpaP with SAG^10^ and this region was used by Esberg *et al*.^13^ for SpaP genotyping, we also zoomed into the amino-acid sequences of this region (residue 385–874). Figure 1b) shows the alignment of the most variable section among the 11 *S. mutans* strains, and Fig. 1c) shows the phylogenetic tree, based on this section. This tree indicates that 11 *S. mutans* strains can be grouped into two clusters. Six *S. mutans* strains are grouped with the type-A strain Nicklas Stromberg 20 with 99–100% sequence similarity within the group, whereas strains UA159, GS-5 and HG723 are grouped with the type-B strain Nicklas Stromberg 40 as their sequences in this region are identical.

### SAG-mediated adherence of *S. mutans* strains

A resazurin metabolic activity assay was used to quantify the SAG-mediated adherence of *S. mutans* strain. A pilot study was carried out using strains UA159 and HG723 to evaluate if the fluorescence intensity (FI) values of the resazurin assay correlate with the viable cell counts of the strains. Cultures in exponential growth phase were serially diluted, and resazurin was added to these dilutions to a final concentration of 0.0016%. Next, the FI values were recorded. For both strains (UA159, HG723), a linear relationship was found between the FI values and CFU/ml counts of each dilution with good correlations (R = 0.99, *p < *0.001) (Supplementary Fig. S1).

**Figure 2:**
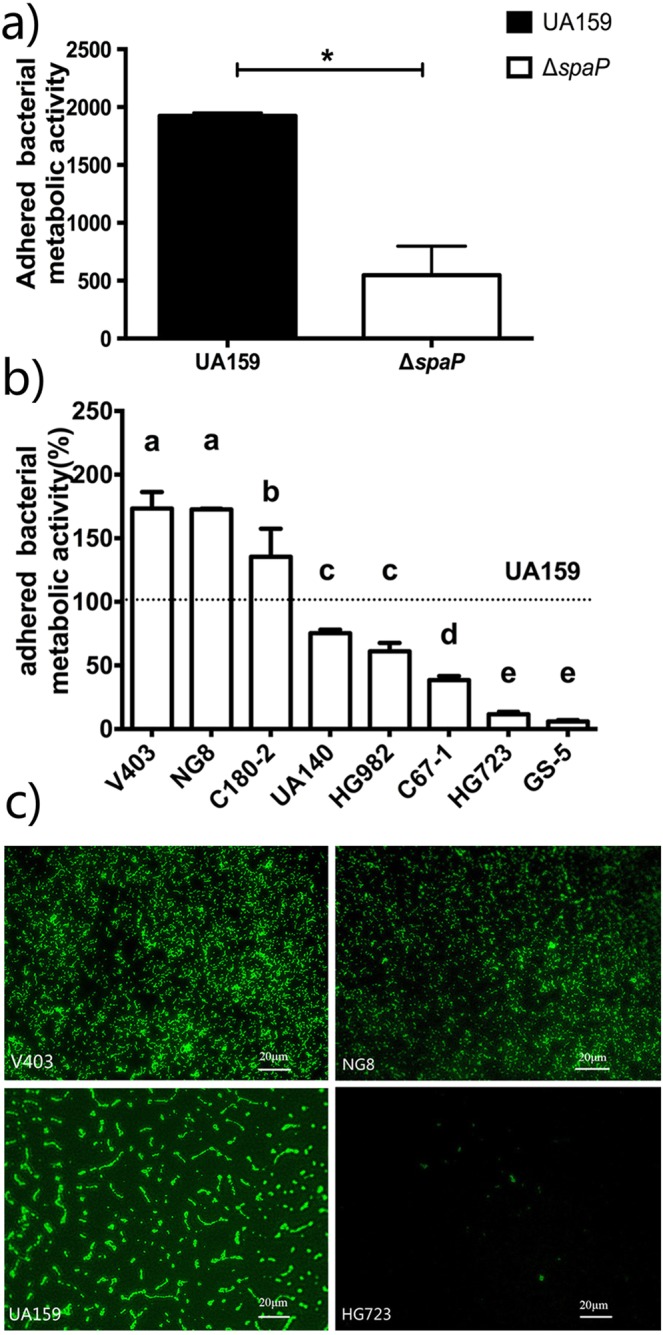
Adherence efficiency of *S. mutans* strains. (**a)** Metabolic activity of adhered cells of UA159 Δ*spaP* compared with UA159. The efficacy of these two strains was evaluated by a resazurin metabolic activity assay. *Statistically significant (*p* < 0.05). (**b)** The percentage of adhered bacteria as measured by metabolic activity in comparison with *S. mutans* UA159. Bars with different letters indicate significant differences (*p* < 0.05). (**c)** Representative images of the *S. mutans* with high, medium and low adherence. The adhered bacteria were stained with a cell-permeable DNA binding fluorescent dye Syto-13.

We first compared the SAG-mediated adherence between *S. mutans* UA159 and its isogenic *spaP* knock out strain. Figure 2a) clearly shows that the knockout strain lost the ability to adhere to the SAG-coated surface, as compared to the wild-type strain. This result confirmed that our assay can be used to examine the SpaP-mediated adherence. Next, we examined the adherence of 9 strains using the same protocol. Figure 2b) demonstrates that the adherence efficiency of *S. mutans* strains varied. Strains V403 and NG8 displayed the highest adherence efficiency, followed by strain C180-2. The adherence efficiency of strain UA140, HG982 and C67-1 was lower than that of strain UA159. The adherence efficiency of strains HG723 and GS-5 was the lowest, only 11% and 6% of the reference strain, UA159.

Figure 2c) shows representative images of the adherence of *S. mutans* strains to SAG-coated coverslips. The images are obtained from the strains with high (strain NG8, V403), medium (strain UA159) and low adherence (strain HG723). Next to the amounts of adhered cells, the pattern of adherence also varied between different strains. The V403 and NG8 cells were in short chains and small aggregates, whereas UA159 cells formed relatively long chains and big aggregates.

### Expression of SpaP on the surface of *S. mutans* cell and in the spent medium

**Figure 3:**
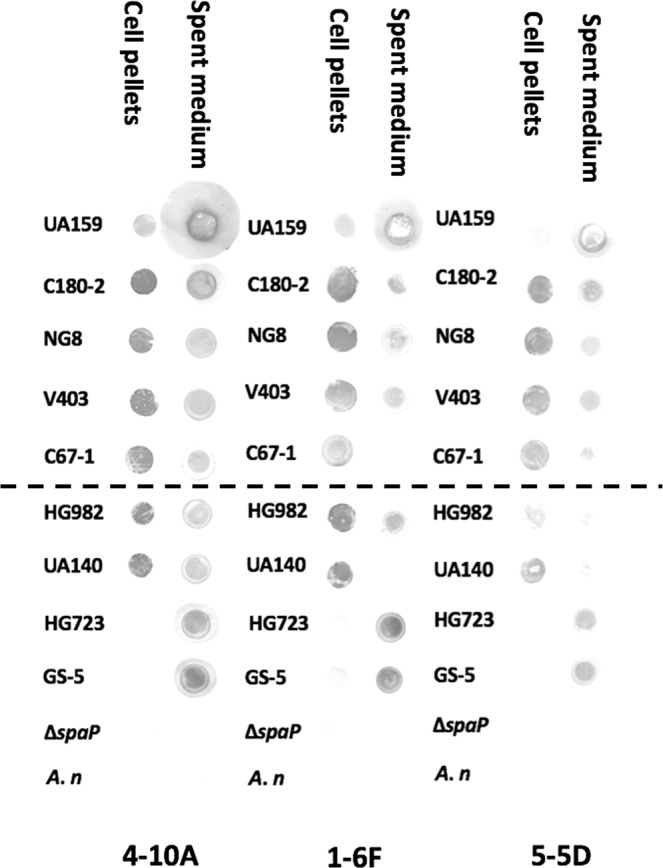
The reactivity of three anti-SpaP mAbs (4-10A, 1-6F, 5-5D) to *S. mutans*. The representative scans from one out of three experiments are shown. The overnight culture of *S. mutans* was adjusted to an OD_600_ of 1.0. Cell pellets and spent-media were incubated with anti-SpaP antibodies 4-10A (the helical stalk of the molecule), 1-6F (globular apical head of the molecule) and 5-5D (A/P interaction at the base stalk of the molecule). *S. mutans* UA159 Δ*spaP* strain (Δ*spaP*) and *Actinomyces naeslundi* ATCC12104 (*A.n*.) were used as negative controls. The original scans of the plots are re-organized for better illustration. The parts below the dash line were next to those above the dash line in the original scans.

A dot-blot assay was performed with anti-SpaP monoclonal antibodies 1-6F, 4-10A, 5-5D of which the approximate binding sites are known (Supplementary Fig. S2)^15^. Neither the cell surface nor the spent media of *S. mutans* UA159 Δ*spaP* and *A. naeslundi* cultures showed antigenicity to the monoclonal antibodies (mAbs). The cell pellets of all (tested) *S. mutans* strains, except HG723 and GS-5, displayed comparable reactivity with mAbs 1-6F that recognizes epitopes within the globular apical head of the molecule^16^ (Fig. 3). Similar to mAb 1-6F, the cell pellets of HG723 and GS-5 did not appear to be reactive to mAb 4-10A and mAb 5-5D (Fig. 3), which map to the helical stalk and the A/P interaction at the base the stalk of the molecule^17^. The cell pellets of strain UA159 shows low antigenicity to the mAbs. In contrast, the spent media of UA159, HG723 and GS-5 displayed a notable reactivity in the antigenicity with 1-6F, 4-10A as well as 5-5D, while the spent media of the other strains showed low antigenicity to these three antibodies.

### Cell surface hydrophobicity of *S. mutans*

The hydrophobicity of mid-log phase cultures of *S. mutans* was determined using hexadecane. Strains HG723, GS-5 and UA159 were considerably less hydrophobic than the other strains (Table 1). Strain UA159 (about 14%) has the lowest hydrophobicity among all tested strains.

**Table 1:** Hydrophobicity of various *S. mutans* strains.

*S. mutans* strain	Mean ± SD
UA140	45.90 ± 2.45^a^
C67-1	44.10 ± 2.67^a^
HG982	42.05 ± 0.60^a^
V403	36.00 ± 2.85^b^
C180-2	35.37 ± 0.70^b^
NG8	34.77 ± 1.93^b^
HG723	27.95 ± 4.11^c^
GS-5	19.09 ± 5.48^d^
UA159	13.79 ± 0.42^e^
UA159 Δ*spaP*	6.28 ± 0.63^f^

Different letters are significantly different from each other (p < 0.05).

## Discussion

*S. mutans* is implicated as a major etiologic agent of dental caries^1^. The interaction between *S. mutans* adhesin SpaP with SAG determines its initial adherence in the oral cavity. Recent studies indicated that *S. mutans* clinical isolates containing *spaP* type-B adhered better to a SAG-coated surface and were associated with a higher caries risk^12,13^. In the current study, we both analyzed the SpaP protein sequences of several known laboratory strains together with our own *S. mutans* clinical isolates and studied the adherence properties of these strains. Based on the sequence of A_3_VP_1_ region of SpaP, we confirmed the previous finding that *S. mutans* strains could be classified into two groups. However, we did not observe any association between the *spaP* genotype and the ability of *S. mutans* to adhere to SAG. In addition, we discovered that besides strain GS-5, strain HG723 also contains a premature stop codon at the C-terminal of SpaP which leads to the loss of adherence to SAG-coated surface of this strain.

In this study, we included a few *S. mutans* laboratory strains, such as the well-known NG8, GS-5 and UA159, because previous knowledge on the structure and functions of SpaP has mostly been obtained from these strains. In addition, these strains have been used for the development and validation of vaccines against *S. mutans*^18–20^. Our data clearly demonstrated that all our strains tested could indeed be classified into the two groups (type-A and type-B) based on the variable V region, which supports the classification of clinical isolates by Esberg *et al*.^13^. Actually, in 1991, Brady *et al*.^21^ already reported that there were two variants of V regions in *S. mutans* SpaP proteins. However, this finding was based on restriction fragment length polymorphism (RFLP) analysis. Different from Esberg *et al*.^13^, we did not observe the enhanced SAG-dependent adherence of the SpaP type B strain as compared to those of the type A strains. Since 2 of the 3 *S. mutans* type B strains showed very low SAG-dependent adherence due to the truncated SpaP proteins, it should be kept in mind that the adherence data in type B group were obtained from one strain (UA159) only. Future studies are needed to screen more *S. mutans* isolates and obtain more type B strains, in order to establish the association between *S. mutans* SpaP genotype and SAG-mediated adherence.

In addition, both this study and the studies of Esberg *et al*.^12,13^. examined the association between SpaP genotypes and the SAG-mediated adherence by analyzing the gene sequences and testing the adherence of the strain on the SAG coated surfaces. With this design, it is impossible to discover if the type of SpaP protein affects its affinity to SAG. Further research is needed to explore this possibility.

Our finding that 2 of the 3 *S. mutans* type B strains showed low SAG-dependent adherence and no SpaP expression on the cell surface indicates that the adherence capability of *S. mutans* depends on the SpaP cell surface expression rather than the genotype of SpaP. Back in 1987, Ayakawa *et al*.^22^ have reported that *S. mutans* strains could be grouped as SpaP retainer and nonretainer strains. In the later studies, it became clear that the SpaP could be retained via different mechanism. The SpaP nonretainer strain NG 5 failed to anchor SpaP to the cell surface due to a point mutation within the *srtA* gene^23^, whereas the other nonretainer strain Ingbritt 162 lost the surface SpaP due to a premature stop codon in SpaP protein. The strain GS-5 was classified to the “others” group in Ayakawa’s study^22^. However, the exact same mutation was discovered in the SpaP of GS-5^24^. It was believed by most researchers that frequent laboratory culturing of the strains led to mutations in an environment lacking appropriate selective pressures^11^. In our study, strain HG723 is our own clinical isolate and has not been cultured frequently. The discovery of the same adenine insertion in this clinical isolate HG723, as in the laboratory strains GS5 and Ingbritt 162, indicates that this mutation may be more frequently present in *S. mutans* strains than previously thought^11^. To exclude the potential contamination of strain HG723 by strain GS-5, we also compared the colony morphology of these two strains on BHI agar (data not shown). They are completely different: the colonies of strain GS-5 are smooth and flat, whereas those of strain HG723 were structured and with flower shape.

Our dot-blot data showed that the cell pellets of strain GS-5 and HG723 did not react with any of SpaP monoclonal antibodies tested, including 1-6F (the globular apical head of the molecule), 4-10A (helical stalk) and 5-5D (A/P region interaction)^17^, whereas the spent medium of these two strains clearly reacted with all tested antibodies. This suggests that the premature termination of SpaP translation in GS-5 and HG723 led to truncated SpaPs, which lack of the C-terminal cell-wall spanning region and the secretion of the protein into the culture medium. Our results were in line with the finding of Jakubovics *et al*.^11^ where the western immunoblot was used to detect the SpaP polypeptides in the spent-medium of strain Ingbritt 162.

It is interesting to observe that the dot-blot expression of the SpaP type-B strain UA159 in spent medium was rather similar to the other two type-B strains (GS-5 and HG723), even though the SpaP of UA159 is complete. The SpaP of strain UA159 in spent-medium seemed to react stronger to the tested antibodies than those on the cell surfaces, whereas the reverse was observed for the type-A strains. Likewise, the SpaP expression on the cell surface of UA159 was much lower than those of the type-A strains. However, the adherence ability of strain UA159 to SAG-coated surfaces was average when compared to other type-A strains. Further research is required to understand the relation between cell-wall localization of SpaP and SAG-mediated adherence in strain UA159.

Cell-surface hydrophobicity was thought to mediate bacterial adherence to teeth and other mucosal surfaces^25^. In our study, the low hydrophobicity of strain GS-5 and HG723 is probably due to the loss of SpaP on their cell surfaces. The hydrophobicity of strain UA159 did not seem to correlate with its adherence to SAG. Its hydrophobicity was the lowest among all tested strains, whereas its adherence ability to SAG was average among all strains. Russell and Smith^26^ found the hydrophobicity of *S. mutans* is associated not only with SpaP, but also with other surface proteins and lipoteichoic acid. Therefore, beside hydrophobic forces, other factors may also contribute to the adherence of *S. mutans*.

In conclusion, the presence of SpaP on the cell surface determined the adherence of *S. mutans* to SAG. With the limited numbers of strains we tested, no relation was found between the genotypes of SpaP and the adherence capability of *S. mutans* strains. Nevertheless, future clinical studies are needed to investigate not only the association between *S. mutans* SpaP genotype and SAG-mediated adherence but also the relationship between the genotype and the caries risk.

## Methods

### Bacterial strains and growth condition

Nine *S. mutans* strains were cultured in this study, including strain UA159 (ATCC 700610, laboratory strain)^27^, UA140 (clinical isolate)^28^, V403 (clinical isolate)^29^, GS-5 (laboratory strain)^24^, NG8 (laboratory strain)^30^, C180-2 (our own clinical isolate), HG723 (our own clinical isolate), HG982 (our own clinical isolate) and C67-1 (our own clinical isolate). All strains were routinely grown on Brain Heart Infusion (BHI) agar or in BHI broth at 37 °C under anaerobic condition (80% N_2_, 10% CO_2_, 10% H_2_). When indicated, erythromycin (Em) was included at 10 μg/ml.

### Comparison of SpaP protein sequences among *S. mutans* strains

Genomic DNA (gDNA) was extracted from overnight culture of 8 out of the cultured 9 *S. mutans* strains using the Genejet Genomic DNA purification kit (Thermo Scientific, MA, USA). The *spaP* gene of an individual *S. mutans* strain was amplified by PCR from the extracted gDNA using a primer set (Supplementary Table S1). The PCR products were purified with a PCR purification kit (Thermo Scientific, MA, USA) and sequenced by Sanger sequencing (GATC Biotech, Constance, Germany). The SpaP amino-acid sequence of *S. mutans* strain NG8 was directly downloaded from NCBI database (accession ACV69919.1). In addition, the sequences of 2 *S. mutans* clinical isolates from Esberg *et al*.^12^ were downloaded (strain Nicklas Stromberg 20; accession MF959050.1 and strain Nicklas Stromberg 40; accession MF959070.1). These two strains belong to type-A and type-B, respectively.

The complete SpaP amino-acid sequences and the A_3_VP_1_-region (residue 385-874) of 11 *S. mutans* strains were aligned in MEGA version 7, using ClustalW with default settings. The phylogenetic trees based on the A_3_VP_1_-region^10^ were also constructed, using the Neighbor-Joining method and 1000 bootstrap replications^31^. Protein similarities were calculated using sequence identity matrix in BioEdit version 7.0.4^32^ using the full SpaP protein alignment.

### SAG-mediated adherence of *S. mutans*

Parotid saliva was collected from healthy volunteers. All volunteers were informed about the objectives and procedure of the study and have signed informed consents before the start of the study. The study was approved by the ACTA ethical review board (201962) and all experiments were carried out in accordance with the guidelines of ACTA. The parotid saliva was collected with a Lashley cup while chewing the sugar-free chewing gum. After collection, the parotid saliva samples were kept on ice for 30 min followed by centrifugation at 4000 × g at 4 °C for 10 min. Subsequently, the supernatant was discarded and the pellets were resuspended with sterile Milli-Q water (crude SAG) by vortexing and pipetting to an equal volume as the original parotid saliva sample.

Crude SAG was diluted 4 times with 0.1 M sodium carbonate (pH = 9.2) and added into the wells of a non-affinity 96-well plate (200 μL/well). This plate was then covered with a lid containing polystyrene pegs (Nunc^TM^, Roskilde, Denmark) overnight at 4 °C to allow the surfaces of pegs to be coated with crude SAG. The pegs were washed on the next day with Phosphate Buffer Saline (PBS) containing 0.1% Tween 20 to remove non-specifically bound proteins.

The individual *S. mutans* strain (9 strains in total) at mid-log phase was used to test adherence to the SAG coated pegs. In detail, *S. mutans* were grown till an OD_600_ of 0.5 in BHI broth and harvested by centrifugation at 5000 × g for 10 min. The cell pellets were washed with adherence buffer (0.05 M KCl, 1 mM CaCl_2_, 1 mM potassium phosphate, 0.1 mM MgCl_2_, pH = 6.0) and adjusted the cell density to 3 × 10^9^ CFU/ml. These *S. mutans* resuspensions were dispensed into a new 96-well plate (200 μL/well). The plate was covered by the lid containing SAG-coated pegs and incubated for 1 h anaerobically at 37 °C. The pegs with adhered *S. mutans* cells were then washed once with PBS and transferred to 0.0016% resazurin solution for 3 h at 37 °C. The metabolic activity of the cells was measured by the changes in fluorescence intensity (FI) of resazurin solutions. The FI was recorded in a spectrofluorometer (Spectramax M2, Molecular Device) with a 485 nm excitation wavelength and 580 nm emission wavelength. For all resazurin measurements, a resazurin solution alone (negative control) was included. The average FI value of the negative control was subtracted from the FI values of the experimental groups before further calculation. The adherence efficiency of each strain was calculated as the percentage relative to the adherence of *S. mutans* UA159. A *spaP* clean knockout strain was constructed. The adherence of *S. mutans* UA159 and the isogenic Δ*spaP* strains (see Supplementary material and Table S1) to SAG-coated surfaces was examined as described above.

In addition, the SAG-mediated adherence of *S. mutans* strain was evaluated with a fluorescence microscope. The Amsterdam Active Attachment (AAA) model^33,34^ containing round glass coverslips (10 mm diameter) was used for the evaluation. Similarly, the coverslips were first coated with crude SAG (1.6 ml/well) overnight at 4 °C and were then washed and transferred to a 24-well plate containing *S. mutans* suspension (1.5 ml/well). The *S. mutans* suspensions were prepared in the same way as above mentioned. After 1 h, the coverslips were washed with PBS and fixed with 4% formaldehyde for 2 h. After fixation, all coverslips were washed twice with PBS and stained with 2 μM SYTO 13 fluorescent nucleic acid staining solution (Invitrogen, Leiden, Netherlands). The images of *S. mutans* adherence were taken with a Zeiss fluorescence microscope (Zeiss, Jena, Germany) with a 40x lens.

In every experiment, each group generally contains 2 replicates and the experiment was repeated 2 times.

### Surface expression of SpaP proteins

A dot-blot assay was used to examine the antigenicity of SpaP protein in the spent-media and on the cell surfaces of 9 *S. mutans* strains. To this end, *S. mutans* overnight cultures were adjusted to a cell density of 3 × 10^9^ CFU/ml and centrifuged at 4000 × g at 4 °C for 10 min. The spent-media and the cell pellets were stored and processed separately. For the spent-medium, protein precipitation was carried out following the method developed by Damerval *et al*.^35^. Four times volume of pre-cooled acetone was added to the spent-medium and kept at −20 °C for 60 min. The precipitated protein was obtained by centrifugation at 12000 × g at 4 °C. The protein pellets were washed and resuspended in PBS to 10 x the initial culture volume. As for the *S. mutans* cell pellets, they were washed and resuspended in PBS.

To carry out the dot-blot assay, the spent-medium protein precipitates or *S. mutans* cell resuspensions (50 μL/sample) were spotted onto a nitrocellulose membrane using Bio-Dot apparatus (Bio-Rad, Hercules, California). The samples were allowed to settle for 30 min before the membrane was removed from the apparatus. The membrane was washed with PBST and probed with mouse anti-SpaP mAb 1-6F_ab_ IgG, 4-10A, 5-5D_6b_^22^ (Kindly provided by Professor L. J. Brady, University of Florida, Gainesville, USA) diluted 1:500 in PBST containing 2% albumin, followed by alkaline phosphatase conjugated goat anti-mouse (1:1000) antibody (Dako, Denmark). The binding sites of anti-SpaP monoclonal antibodies mapped on the tertiary structure of SpaP are shown in Supplementary Fig. S2. For visualization blots with alkaline phosphatase were stained with 5-bromo-4-choro-3 indolyl phosphate (Roche Diagnostics, Mannheim, Germany). *S. mutans* UA159 Δ*spaP* strain and *Actinomyces naeslundi* ATCC12104 which do not contain the SpaP protein were used as negative control strains.

### Hydrophobicity of *S. mutans*

The hydrophobicity of *S. mutans* strains was evaluated with hexadecane, as described by Rosenberg *et al*.^36^. In brief, *S. mutans* cells grown till mid-log phase were harvested and resuspended in PBS to a final OD_600_ of 0.5. To every 3 ml resuspension, a volume of 150 μl hexadecane (Sigma, Damstadt, Germany) was added. The mixture was vortexed for 10 s and then settled for 20 min at room temperature before the OD_600_ value of the aqueous layer was determined. Hydrophobicity was expressed as the percentage of reduction in OD_600_ of the aqueous phase relative to that of the initial resuspension.

### Statistical analysis

The data were analyzed with the Statistical Package for Social Science (SPSS, Version 23.0, Chicago, IL, USA). *Student’s t*-test was applied to examine the difference of adhered bacterial metabolic activity between *S. mutans* UA159 and *S. mutans* UA159 Δ*spaP*. One-way ANOVA was used to evaluate the difference in adherence and hydrophobicity among the *S. mutans* strains, followed by Student-Newman-Keuls post-hoc test. Differences were considered statistically significant at *p* < 0.05.

## Supplementary information

Supplementary Dataset 1
