# Supplementary material for: Diversity of SpaP in genetic and salivary agglutinin mediated adherence among Streptococcus mutans strains
Source: Sci Rep. 2019 Dec 27;9:19943. doi: 10.1038/s41598-019-56486-9 (PMC6934801; doi:10.1038/s41598-019-56486-9)
Supplement: Supplementary file 1 — Supplementary Dataset 1 [file 41598_2019_56486_MOESM1_ESM.docx]

**Diversity of SpaP in genetic and salivary agglutinin mediated adherence among *Streptococcus mutans* strains**

Jingmei Yang^1,2^, Dongmei Deng^2^, Bernd W Brandt^2^, Kamran Nazmi^3^, Yafei Wu^1^, Wim Crielaard^2^, Antoon JM Ligtenberg^3^

1. State Key Laboratory of Oral Diseases & National Clinical Research Center for Oral diseases & Department of Periodontics, West China School & Hospital of Stomatology, Sichuan University, Chengdu, China
2. Department of Preventive Dentistry, Academic Centre for Dentistry Amsterdam, University of Amsterdam and Vrije Universiteit Amsterdam, Amsterdam, the Netherlands
3. Department of Oral Biochemistry, Academic Centre for Dentistry Amsterdam, University of Amsterdam and Vrije Universiteit Amsterdam, Amsterdam, the Netherlands

**Supplementary Material**

### Construction of *S. mutans* UA159 Δ*spaP* strain

The *S. mutans* UA159 Δ*spaP* strain was constructed using a precise deletion method ^33,37^. Briefly, a crossover PCR deletion product was obtained in two steps: (i) the upstream and downstream fragments of *spaP* gene were generated with primer pairs *spaP*uf/*spaP*ur and *spaP*df/*spaP*dr respectively, using chromosomal DNA of strain UA159 as a template; (ii) the two fragments were annealed at their overlapping region. The resulting fragment was amplified with primers *spaP*uf/*spaP*dr. The new fragment, containing upstream and downstream of *spaP* gene, were ligated into a suicide vector pORI280. The new vector was transformed into *S. mutans* UA159 and selected on BHI plates containing Em. The correct transformant was grown in BHI broth without antibiotics for 30-40 generations and plated on BHI agar plates containing 100μg/ml 5-bromo-4-chloro-3-indolylgalactopyranoside (X-gal). The white colonies (excluded vector fragments) were selected. The deletion of *spaP* gene and the correct up and down sequences of the *spaP* gene were verified by Sanger sequencing (GATC Biotech, Constance, Germany).

**Table S1. Primers used in this study**

|  | Primer sequence (5’-3’) |
| --- | --- |
| *spaP*uf | TCGTGAGAATTCAAGAGCAGCCAAGGAT |
| *spaP*ur | AACTAAGCCACCGTAAGTTTTTTTGACTTTC |
| *spaP*df | ACTTACGGTGGCTTAGTTACTAGTTTTAGTTT |
| *spaP*dr | CTAAGTGCATGCTGTGCTTGACCAATAA |
| *spaP*F | ATGAAAGTCAAAAAAACTTAC |
| *spaP*R | ATAAAAGCTCAATCTGTGAT |

**Figure S1**

Figure S1. The relationship between fluorescent intensity in resazurin assay and viable counts in *S. mutans* UA159 and HG723. (A) FI values of *S. mutans* UA159 at 2 hours were plotted against the viable cell counts. (B) FI values of *S. mutans* HG723 were plotted against the viable cell counts.

**Figure S2**


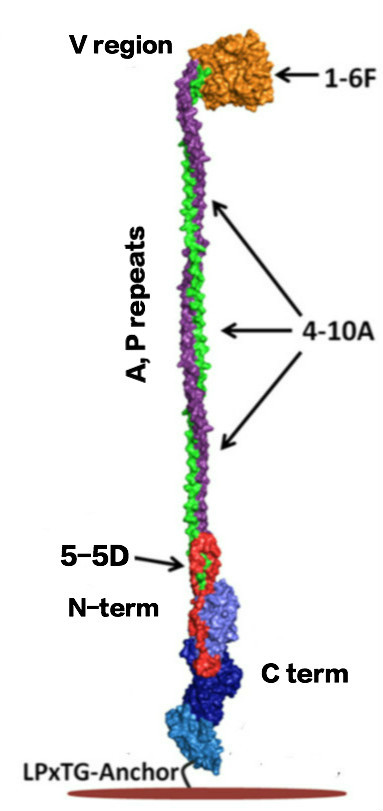


Figure S2. The binding sites of anti-SpaP monoclonal antibodies used in this study ^17^.

**References**

[37] Leenhouts, K. *et al*. A general system for generating unlabelled gene replacements in bacterial chromosomes. *Mol Gen Genet* **253**, 217–224 (1996).
